# Legacy Effects of *Urochloa brizantha* Cover Cropping on Rhizosphere Fungal Communities and Soil Properties in a Degraded Common Bean System

**DOI:** 10.3390/jof12070456

**Published:** 2026-06-23

**Authors:** Carla Luciana Abán, Giovanni Larama, Antonella Ducci, Ana Fallard, Javier Ortiz, Silvina Vargas-Gil, Carolina Pérez-Brandan

**Affiliations:** 1 Consejo Nacional de Investigaciones Científicas y Técnicas (CONICET), Salta 4400, Argentina; aban.carla@inta.gob.ar (C.L.A.); antonelladucci92@gmail.com (A.D.); 2 Estación Experimental Agropecuaria Salta, Instituto Nacional de Tecnología Agropecuaria (INTA), Cerrillos, Salta 4400, Argentina; 3 Biocontrol Research Laboratory, Universidad de La Frontera, Temuco 4780000, Chile; giovanni@larama.cl; 4 Biome Quest SpA, Villarrica 4930000, Chile; 5 Instituto de Investigaciones Agropecuarias (INIA Carillanca), Km 10, Camino Cajón-Vilcún, Vilcún 4940000, Chile; 6 Laboratorio de Biorremediación, Facultad de Ciencias Agropecuarias y Medioambiente, Universidad de La Frontera, Temuco 4780000, Chile; javier.ortiz@ufrontera.cl; 7 Unidad de Fitopatología y Modelización Agrícola (UFYMA-CONICET), Instituto de Patología Vegetal (IPAVE), Centro de Investigaciones Agropecuarias (CIAP-INTA), Córdoba 5000, Argentina; vargasgil.silvina@inta.gob.ar

**Keywords:** *Urochloa brizantha*, metagenomics, cover crops, soil health

## Abstract

Intensive agricultural practices based on continuous monocropping and prolonged bare-soil fallows have contributed to soil degradation and loss of biological functioning. Replacing fallows with cover crops (CCs) is a promising strategy to restore soil quality, yet their legacy effects on rhizosphere fungal communities remain poorly understood. This study evaluated the legacy effects of *Urochloa (syn. Brachiaria) brizantha* cover cropping on rhizosphere fungal communities, as well as soil physicochemical and biological properties, in a degraded common bean system. A field experiment with a randomized complete block design included: bare fallow (BM), one (B1) or two (B2) CC cycles before bean, a perennial pasture (PB), and a pristine soil reference (PS). High-throughput sequencing showed that Urochloa-based treatments significantly shifted fungal community composition compared to BM, increasing saprotrophic and beneficial taxa (e.g., *Mortierella*, *Penicillium*, *Coprinellus*) and reducing potential pathogens such as *Fusarium*. These changes were associated with higher soil organic carbon, aggregate stability, microbial biomass, and enzyme activities, especially in B2 and PB. Indicator taxa identified by LEfSe were linked to organic matter decomposition and nutrient cycling. Multivariate analyses revealed strong associations between fungal community structure and soil properties. Overall, *U. brizantha* cover cropping induced measurable legacy effects, promoting soil biological recovery even after short-term implementation.

## 1. Introduction

Intensive agricultural practices, including monoculture, excessive tillage, and the overuse of chemical inputs, have contributed to widespread soil degradation, loss of biodiversity, and declines in fertility [1]. In long-term cultivated agroecosystems, these processes compromise vital physical properties and ecological functions, such as water balance and nutrient cycling, ultimately threatening the sustainability of food production [2]. To mitigate these impacts and restore soil health, it is essential to understand how conservation practices, such as cover crops, generate legacy effects that can accelerate ecosystem recovery. Consequently, restoring soil ecological functioning while maintaining crop productivity has become a major goal of sustainable agriculture [3].

Soil microorganisms are fundamental drivers of soil ecosystem functioning, contributing to organic matter turnover, nutrient cycling, soil aggregation, and plant health [4,5]. Among them, fungi are key drivers of soil structure and nutrient dynamics due to their ability to decompose complex organic substrates and form extensive hyphal networks [6,7,8]. These interactions are especially pronounced in the rhizosphere, the narrow soil zone strongly influenced by root exudation and rhizodeposition [9]. As a dynamic interface, the rhizosphere represents a hotspot of biological activity where microbial communities are actively selected by plants and, in turn, regulate nutrient availability, plant growth, and defense against soil-borne pathogens [5,10,11]. Recent studies indicate that rhizosphere microbial communities are shaped not only by the current host plant, but also by antecedent vegetation and management history, generating soil legacy effects that persist across cropping cycles [12,13,14,15]. Through plant–soil feedback mechanisms, management-driven shifts in microbial communities can modify soil physicochemical and biological properties, which subsequently influence soil functioning and plant–soil interactions in subsequent crops [16,17,18]. Within this context, cover crops (CCs), are key components of sustainable agricultural systems [19,20]. CCs can enhance soil structure, improve nutrient cycling, increase soil organic matter inputs, suppress weeds, and support diverse and functionally active soil microbial communities. Among them, grasses of the genus *Urochloa* (syn. *Brachiaria*) are particularly promising due to their high biomass production, extensive root systems, and adaptability to low-fertility soils [21].

In South America, *Urochloa* species are widely used in livestock systems and have increasingly been integrated into cropping systems or in crop–livestock rotations, where they improve soil quality properties [22,23,24,25]. While several studies have described microbial communities associated with *Urochloa* spp. roots and residues, their legacy effects on the rhizosphere microbiome of subsequent crops remain poorly understood [26,27,28]. In the main common bean-producing region of Argentina, agricultural systems are predominantly based on continuous monocropping, prolonged bare-soil fallows, limited crop rotation, and intensive agrochemical use, leading to soil organic matter losses, reduced aggregate stability, and increased incidence of soil-borne diseases [29,30]. The transition toward sustainable agricultural practices is not merely a strategy for maintaining productivity, but a critical pathway for global ecological resilience and the restoration of ecosystem functions in degraded landscapes [31]. In this regional context, the integration of *U. brizantha* as a cover crop during fallow periods represents a promising strategy to restore soil ecological functioning in degraded agroecosystems. The novelty of this study lies in unraveling how these management-driven footprints actively shape the fungal community assembly in the rhizosphere of the common bean. By evaluating a temporal gradient of exposure (one versus two consecutive cycles) in a system with over 50 years of intensive agricultural degradation, this research identifies whether even short-term interventions can initiate measurable ecological recovery trajectories through shifts in fungal structure and functional potential [32].

Therefore, the objective of this study was to evaluate the legacy effects associated with the inclusion of *U. brizantha* during fallow periods by assessing rhizosphere soil properties and the root-associated fungal community under different durations of cover crop exposure. We hypothesized that the inclusion of *U. brizantha* during fallow periods would modify soil conditions, driving shifts in the rhizosphere of the subsequent crop fungal community structure and functional potential.

## 2. Materials and Methods

### 2.1. Experimental Design

**Figure 1:**
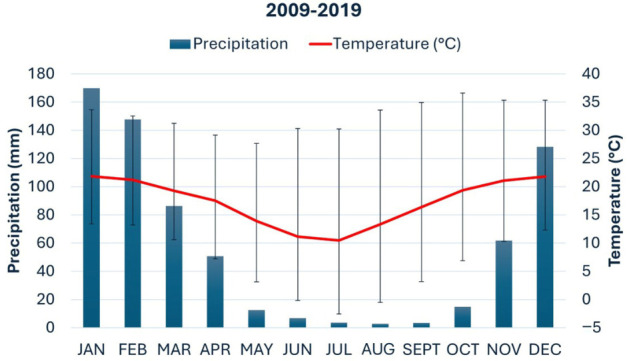
Monthly precipitation (bars) and mean temperature (line) with error bars showing the range of minimum and maximum temperatures during the period 2009–2019.

A medium-term field trial (2009–2019) was conducted at the Salta Agricultural Experimental Station of the National Agricultural Technology Institute (EEA-INTA) in Cerrillos, Salta, Argentina (S 24°53′52.84″; W 65°27′59.11″, 1420 m.a.s.l.). The region is characterized by a subtropical climate with a pronounced dry winter and a rainy summer, with most annual precipitation concentrated between November and March. Mean annual precipitation and temperature during the study period are presented in Figure 1. The soil type of the region is predominantly loam with 1.31% organic matter (32% sand, 44% silt, 24% clay), Ustocrepte Udico (USDA Soil Taxonomy) soil, Cerrillos series with A, AC, and C horizons. Before trial establishment, the study site had been subjected to more than five decades of intensive agricultural use under conventional management. The system was initially dominated by tobacco cultivation, a crop historically associated with frequent soil disturbance and intensive tillage practices. Then, the system was later replaced by long-term common bean monocropping. Together with extended fall–winter fallow periods, this prolonged history of intensive management led to a degraded soil condition at the onset of the trial, characterized by reduced soil quality and biological functioning. A detailed description of the historical management of the site is provided in Abán et al. [32].

The field experiment followed a randomized complete block design (RCBD) with three replicates. Each plot measured 15 × 50 m and constituted an independent experimental unit. The treatments were: BM: bare-soil fallow followed by common bean; B1: one cycle of *U. brizantha* cover cropping before common bean; B2: two consecutive cycles of *U. brizantha* cover cropping before common bean; PB: a perennial *U. brizantha* pasture. A pristine soil (PS) was included as an external, non-cropped reference. Treatments B1, B2 and BM were previously described in detail in previous studies [30]. Briefly, in BM, common bean was cultivated as a continuous monoculture under conventional management, with long fall–winter fallow periods between crop cycles and no fertilizer application. In treatments B1 and B2, *U. brizantha* was used as a cover crop during the fallow period. In B1, *U. brizantha* was sown in September during the fallow period, and at the end of November, during flowering, the grass was chemically desiccated prior to common bean sowing. In B2, *U. brizantha* was maintained as pasture for two consecutive crop cycles before common bean reintroduction and then desiccated before common bean planting. No fertilizers, pesticides, or other agrochemicals were applied during the pasture phase. In PB, *U. brizantha* was established in 2009 and maintained continuously as a perennial pasture without further management, serving as a reference for soil recovery under permanent plant cover. Finally, pristine soil (PS) was sampled as a reference site to establish a baseline for soil properties in the absence of anthropogenic disturbance. These soil samples were located approximately 50 m from the experimental field, adjacent to the agricultural treatments. Unlike PB, PS was not part of the experimental design and was only used as an external reference.

### 2.2. Soil Sampling

Soil sampling was performed at the flowering stage (R5) of common bean plants at the end of the field experiment in 2019. A total of 12 composite samples were collected from the experimental plots (4 treatments × 3 replicates), plus 3 additional composite samples from the reference site. For each replicate, composite samples were collected by removing 15 common bean plants with a shovel to a depth of 10 cm.

First, loosely bound soil (acting as the outer rhizosphere compartment) adhering to the roots was collected by vigorously shaking the roots by hand, then sieved (2 mm), homogenized, and subdivided for physicochemical and microbiological analyses. Then, one subsample was air-dried (20 ± 2 °C for 24 h) for physical and chemical characterization, while the other was stored at 4 °C for microbial biomass and enzymatic activity measurements. In addition, tightly bound rhizosphere soil, defined as the soil layer directly adhering to the root surface and influenced by root exudates, was collected by gently brushing the root surface and stored at −20 °C for DNA extraction and molecular analyses [32].

### 2.3. Soil Chemical, Physical, and Microbiological Analyses

Soil organic carbon (SOC) and organic matter (OM) were determined by the wet oxidation method of Walkley and Black [33]. Total nitrogen (TN) was quantified using the micro-Kjeldahl method [34]. Extractable phosphorus (eP) was determined according to the Bray and Kurtz method [35]. Exchangeable cations, including sodium (Na^+^), potassium (K^+^), calcium (Ca^2+^), and magnesium (Mg^2+^), were determined using an atomic absorption spectrometer (Perkin Elmer 5100 PC, Shelton, CT, USA).

Soil physical properties included bulk density (BD), measured using the core method described by Blake and Hartge [36], and aggregate stability (AS), determined using the wet-sieving method of Corvalán et al. [37]. Water-holding capacity (WHC) was determined gravimetrically, while soil pH and electrical conductivity (EC) were measured potentiometrically in a 1:2.5 soil-to-water suspension.

Microbial biomass carbon (MBC) and nitrogen (MBN) were determined using the chloroform fumigation–extraction method [38], and microbial respiration (MR) was determined as CO_2_–C evolution, following Alef [39]. Easily extractable glomalin (GRSP/EEG) was obtained from 1 g of sieved rhizosphere soil using citrate extraction, autoclaving and centrifugation, and the protein content in the supernatant was quantified by the Bradford assay with bovine serum albumin as the standard [40]. Enzymatic activities associated with overall microbial activity were assessed by fluorescein diacetate hydrolysis (FDA) [41] and dehydrogenase activity (DHA) [42]. Acid phosphatase activity (AP) was determined following Tabatabai and Bremner [43].

### 2.4. DNA Extraction, Amplicon Sequencing and Bioinformatic Processing

DNA was extracted from 0.250 g of rhizosphere soil using a PowerSoil^®^ DNA Isolation kit (Qiagen, Hiden, Germany) following the manufacturer’s instructions. The quality and quantity of the DNA extracted were determined using a DeNovix DS-11 spectrophotometer (DeNovix, Wilmington, DE, USA) and by agarose gel electrophoresis (1.0%).

Fungal communities were analyzed using PacBio amplicon sequencing targeting the full-length internal transcribed spacer (ITS) region, a standard DNA barcode for fungi [44]. Amplification was performed at the Integrated Microbiome Resource (IMR), Dalhousie University, Halifax, Nova Scotia, Canada, using the high-coverage primer set ITS1F_KYO2 (5′-TAGAGGAAGTAAAAGTCGTAA-3′) and ITS4_KYO1 (5′-TCCTCCGCTTWTTGWTWTGC-3′) [45]. The PCR amplification was carried out under the following thermocycling conditions: an initial denaturation step at 94 °C for 1 min; followed by 35 cycles consisting of denaturation at 94 °C for 30 s, primer annealing at 47 °C for 30 s, and extension at 68 °C for 30 s; with a final extension step at 68 °C for 10 min, before being held at 4 °C. All reactions were performed in triplicate to reduce amplification bias, pooled, and verified via agarose gel electrophoresis prior to purification and sequencing. High-accuracy circular consensus (HiFi) reads were generated to obtain full-length ITS sequences. Sequence processing and amplicon sequence variant (ASV) inference were conducted using DADA2, including quality filtering, denoising, and chimera removal [46]. Taxonomic assignment was performed in QIIME 2 using a Naive Bayes classifier trained on the UNITE fungal database (version 10) [47,48].

### 2.5. Statistical and Bioinformatic Analyses

Soil chemical, physical, and microbiological variables were analyzed using analysis of variance (ANOVA) considering a randomized complete block design, with management treatment as a fixed effect and block as a random effect. When treatment effects were significant (*p* < 0.05), mean comparisons were performed using Fisher’s least significant difference (LSD) test. All statistical analyses were conducted using R software (version 4.1.1), primarily with the stats and agricolae packages.

Although a sample size of three biological replicates per treatment may constrain the statistical power of PERMANOVA and LEfSe, field-scale variance and spatial heterogeneity were strictly controlled using a randomized complete block design (RCBD). Alpha diversity was estimated using the Shannon diversity index, Pielou’s evenness, and observed features, calculated on samples rarefied to an even depth of 3322 reads (minimum library size). Beta diversity was assessed using Aitchison distance based on centered log-ratio (CLR)-transformed ASV abundances, accounting for data compositionality [49]. Differences in community composition among groups were tested using PERMANOVA with 1000 permutations [50]. Indicator taxa were identified using Linear discriminant analysis Effect Size (LEfSe), with an LDA score threshold of 2.0 and α = 0.05. Genus-level biomarkers identified by LEfSe were independently validated using a zero-inflated Gaussian mixture model (ZIGMM) implemented in metagenomeSeq through the microbiomeMarker package, applying Benjamini–Hochberg correction for multiple comparisons (Supplementary Table S1). Functional potential was inferred by assigning ASVs to ecological guilds with the FUNGuild database [6]. To ensure accuracy, only functional guilds with a confidence ranking of ‘highly probable’ or ‘probable’ were retained for downstream analysis, discarding any ‘possible’ assignments. Differential abundance of fungal guilds among treatments was evaluated using the DESeq2 R package [51], based on negative binomial generalized linear models with Wald tests and Benjamini–Hochberg correction for multiple testing; guilds with adjusted *p* < 0.05 were considered significantly differentially abundant. Relationships between fungal community structure and physicochemical and microbiological variables were examined using redundancy analysis (RDA) implemented in the vegan R package, with statistical significance assessed by permutation tests [52,53]. To further evaluate the relative contribution of biological and physicochemical variables, partial redundancy analyses were performed. Environmental variables were grouped into biological (GRSP, MR, MBN, MBC) and physicochemical (Mg, WHC, eP, C:N) categories. Partial RDAs were performed to quantify the unique and shared fractions of variance explained by each group, and adjusted R^2^ values were reported to account for model complexity. Pairwise associations between dominant fungal genera and environmental variables were examined using Spearman’s rank correlation coefficients using the *corrplot* version 0.84 package in R [54]. Although the pristine soil (PS) was sampled externally and was not part of the randomized complete block design, it was intentionally included in the global inferential and multivariate statistical analyses (ANOVA, PERMANOVA, LEfSe, and RDA). This approach was utilized to establish a robust ecological baseline under a unified error variance, allowing for direct multi-treatment comparisons to assess whether the legacy effects of the *Urochloa brizantha* treatments significantly shift the degraded system back toward the configuration of an undisturbed ecosystem.

## 3. Results

### 3.1. Sequencing Results and Diversity Analysis

The sequencing run produced 165,689 raw reads across 15 input libraries (Supplementary Table S2). After primer removal, filtering, and denoising, 75–80% of reads were retained on average, resulting in ~7500 high-quality sequences per sample. After chimera removal, 122,805 non-chimeric reads were retained, corresponding to ~77% of the raw sequences. Alpha diversity of the fungal community did not differ significantly among treatments, as indicated by Kruskal–Wallis tests for Shannon diversity (H = 5.33, *p* = 0.255), evenness (H = 6.10, *p* = 0.192), and observed features (H = 3.43, *p* = 0.488) (Figure S1).

**Figure 2:**
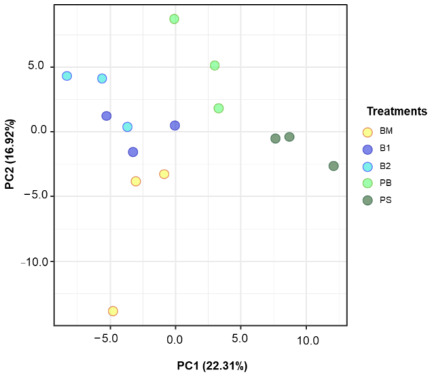
Principal component analysis of fungal communities based on the Aitchison distances measured in the rhizosphere of common bean following different cover crop treatments (*n* = 3): BM, bare-soil fallow followed by common bean; B1, one cycle of *U. brizantha* cover cropping before common bean; B2, two consecutive cycles of *U. brizantha* cover cropping before common bean; PB, a perennial *U. brizantha* pasture; PS, Pristine soil under native vegetation, included as a reference site.

PERMANOVA analysis revealed that fungal communities differed significantly across treatments (R^2^ = 0.378, F = 2.0, *p* < 0.001) (Figure 2). According to PC1 (22.31%), the fungal communities of B1 and B2 had a high similarity among each other, with a slight separation between them. In addition, B1 and B2 were positioned close to BM. The PB samples occupied an intermediate position between the cover crop treatments and the pristine site, while the PS samples were located separately to the right, clearly distinct from all managed systems. Along PC2 (16.92%), the BM treatment was clearly separated from the other treatments, highlighting a distinct fungal community, whereas B1, B2, PB, and PS remained on the opposite side of this axis, highlighting the marked effect of the monoculture system.

### 3.2. Fungal Community Composition

**Figure 3:**
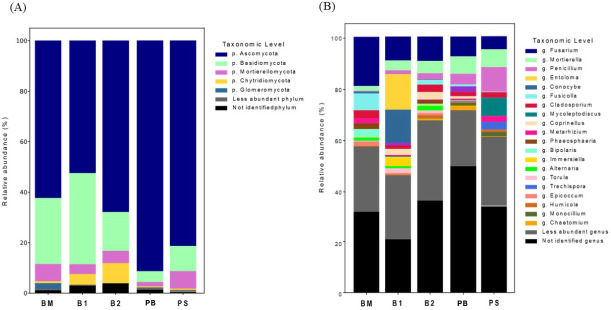
Average of relative abundance at phyla (**A**) and genera (**B**) taxonomic levels in the total fungal community associated with common bean rhizosphere following different cover crop treatments: BM, bare-soil fallow followed by common bean; B1, one cycle of *U. brizantha* cover cropping before common bean; B2, two consecutive cycles of *U. brizantha* cover cropping before common bean; PB, a perennial *U. brizantha* pasture; PS, Pristine soil under native vegetation, included as a reference site.

The relative abundance of fungal taxa differed among treatments (Figure 3A). At the phylum level, *Ascomycota* dominated all treatments followed by *Basidiomycota* (4–36%), *Mortierellomycota* (2–7%), *Chytridiomycota* (<7%) and *Glomeromycota* (<3%)*. Ascomycota* dominated all treatments, particularly in PB and PS, while *Basidiomycota* was more abundant in treatments under *U. brizantha* and BM. *Mortierellomycota* were relatively enriched in PS, in treatments under *U. brizantha,* and in BM, *Chytridiomycota* were most frequent in B1 and B2, and *Glomeromycota* appeared mainly in BM.

At genus level, *Fusarium* was consistently present across treatments, with the highest abundance in BM (19.3%) (Figure 3B). *Mortierella* was relatively enriched in PB (6.8%) and PS (6.9%), while *Penicillium* was most abundant in PS (9.58%) and lowest in BM (0.45%). Some genera such as *Entoloma* and *Conocybe* were mainly detected in B1 (13.8% and 12.8%, respectively), whereas *Fusicolla* reached higher abundance in BM (6.61%) than the rest of the treatments. Other genera, including *Cladosporium*, *Chaetomium*, and *Monocillium*, were detected at lower proportions across treatments (≤3%).

### 3.3. LEfSe Analysis

**Figure 4:**
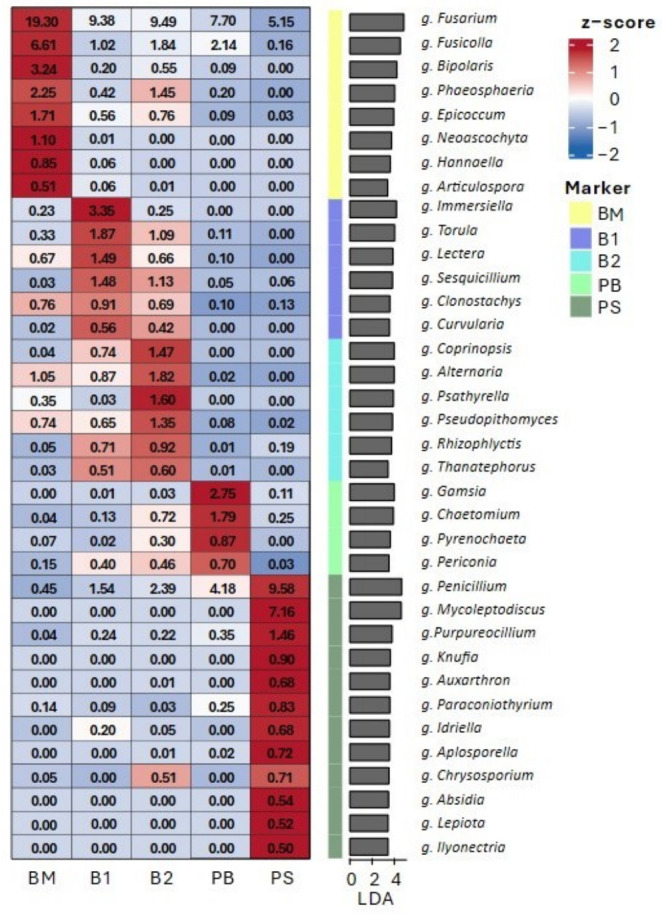
Differential abundance of fungal taxa in the rhizosphere samples identified by LEfSe analysis following different cover crop treatments: BM, bare-soil fallow followed by common bean; B1, one cycle of *U. brizantha* cover cropping before common bean; B2, two consecutive cycles of *U. brizantha* cover cropping before common bean; PB, a perennial *U. brizantha* pasture; PS, Pristine soil under native vegetation, included as a reference site. Relative abundance of each genus is shown and color-coded according to standardized Z-scores, with red indicating enrichment (positive values) and blue indicating depletion (negative values) relative to the overall mean abundance across treatments. Grey bars represent LDA scores (log_10_), indicating the effect size of each taxon as a discriminant feature among treatments.

The linear discriminant analysis effect size (LefSe) revealed treatment-specific fungal biomarkers in the rhizosphere across the different management treatments (Figure 4). At the genus level, LEfSe analysis identified a total of 36 biomarker genera, with 8 biomarkers in BM, 6 in B1, 6 in B2, 4 in PB, and 12 in PS (Figure 4). Among the most representative genera, BM showed enrichment of *Fusarium*, *Fusicolla*, and *Bipolaris*. B1 was mainly associated with *Immersiella*, *Torula*, and *Lectera*, while *Coprinopsis*, *Alternaria*, and *Psathyrella* were the most representative genera in B2. PB showed enrichment of *Gamsia*, *Chaetomium*, and *Pyrenochaeta*. Finally, PS was characterized by *Penicillium*, *Mycoleptodiscus*, *Purpureocillium* and *Knufia*. These biomarkers were independently validated using a conservative, model-based approach (ZIGMM; Supplementary Table S1). Most of the LEfSe biomarkers were corroborated by ZIGMM in the same direction, including *Fusicolla* in BM (adjusted *p* = 0.045), with the exception of *Fusarium*, which was not statistically significant under this model.

### 3.4. Functional Potential of Rhizosphere Fungal Communities

**Figure 5:**
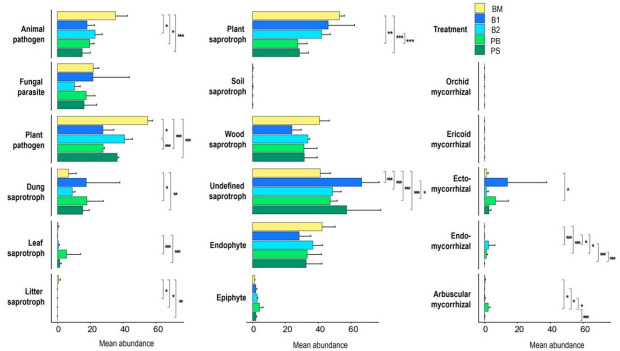
Relative abundance of fungal guilds following different cover crop treatments: BM, bare-soil fallow followed by common bean; B1, one cycle of *U. brizantha* cover cropping before common bean; B2, two consecutive cycles of *U. brizantha* cover cropping before common bean; PB, a perennial *U. brizantha* pasture; PS, Pristine soil under native vegetation, included as a reference site. Asterisks denote the level of statistical significance: *p* < 0.05 (*), *p* < 0.01 (**), and *p* < 0.001 (***).

Functional guild (FUNGuild) analysis showed that the most abundant guilds included plant pathogens, plant saprotrophs, wood saprotrophs, endophytes, and undefined saprotrophs, which together accounted for the majority of the community in all treatments (Figure 5). Notably, a considerable proportion of the sequences across all management systems were classified as undefined saprotrophs, which highlights a recognized limitation of the FUNGuild database in fully resolving environmental fungal sequences lacking species-level ecological characterization. In particular, the BM treatment exhibited a higher relative abundance of plant pathogens and plant saprotrophs, whereas B1 was characterized by a greater contribution of undefined saprotrophs and a comparatively lower representation of plant pathogens. B2 displayed intermediate values, maintaining relatively high saprotrophic abundance but with lower representation of plant-associated pathogenic guilds compared to BM. Perennial *U. brizantha* (PB) and pristine soil (PS) treatments were characterized by a shift toward more balanced functional profiles, with lower relative abundance of plant pathogens and a greater contribution of saprotrophic and symbiotrophic guilds.

### 3.5. Effect of U. brizantha on Chemical, Physical and Microbiological Soil Properties

**Figure 6:**
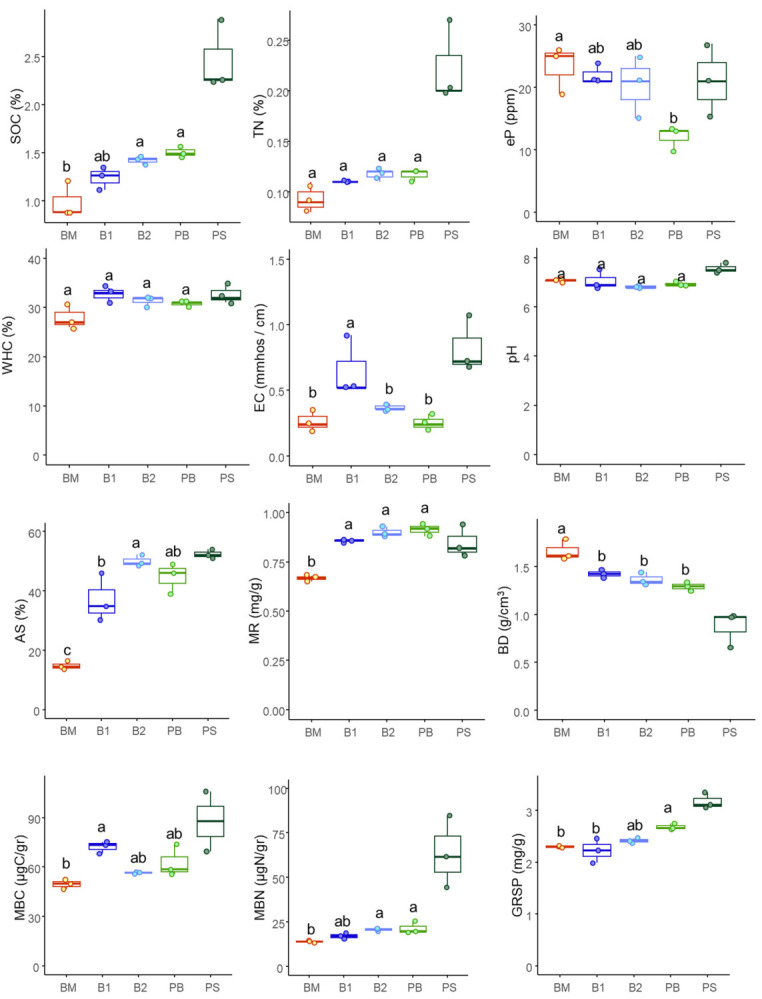
Mean values of soil chemical, physical and microbiological properties measured in the rhizosphere following different cover crop treatments: BM, bare-soil fallow followed by common bean; B1, one cycle of *U. brizantha* cover cropping before common bean; B2, two consecutive cycles of *U. brizantha* cover cropping before common bean; PB, a perennial *U. brizantha* pasture; PS, Pristine soil under native vegetation, included as a reference site. Different letters indicate values that are significantly different (*p* < 0.05). Colors represent the different treatments: BM (red), B1 (blue), B2 (light blue), PB (light green), and PS (dark green).

The chemical, physical, and microbiological properties of soil responded to the inclusion of *Urochloa brizantha* as a cover crop or pasture (Figure 6). SOC, as the main component of soil organic matter, indicates the lowest values under common bean monoculture without CC (BM), while treatments including *U. brizantha* showed higher values, particularly under B2 and PB. In contrast, total nitrogen (TN) did not differ significantly among treatments, although low levels were observed in BM. Extractable phosphorus (eP) was similar among treatments, except for PB, which exhibited significantly lower values compared to BM.

Regarding soil physical properties, water-holding capacity (WHC) did not differ significantly among treatments; however, it was lower in BM (Figure 6, Supplementary Table S3). Soil electrical conductivity (EC) increased significantly only in B1, whereas values under the remaining *U. brizantha* treatments were comparable to those observed under BM. No significant differences were observed in soil pH among the treatments. Aggregate stability was lowest under BM and increased significantly under *U. brizantha* treatments, reaching values comparable to the reference site. Bulk density was highest under BM, while treatments with *U. brizantha* showed lower and similar levels.

Microbiological indicators also varied in response to management. Microbial respiration was significantly higher under *U. brizantha* treatments, with values comparable to the reference site. Microbial biomass C and N varied among treatments, with the lowest values consistently observed under BM. Higher MBC values were observed in B1, while MBN was higher in B2 and PB, whereas the remaining treatments showed intermediate responses. The glomalin-related soil protein (GRSP) content increased significantly under perennial *U. brizantha* (PB), compared to BM and B1, which showed similarly low values, whereas B2 exhibited intermediate levels.

**Figure 7:**
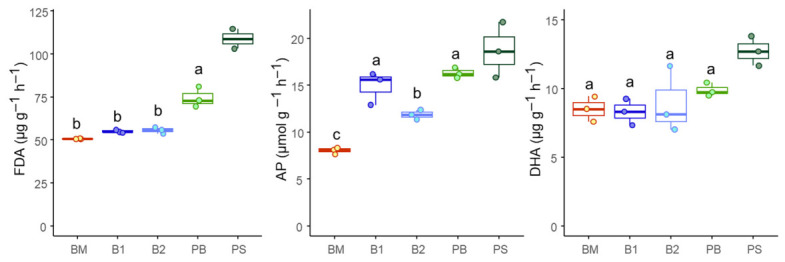
Mean values of fluorescein diacetate (FDA) hydrolysis, acid phosphatase (AP) activity and dehydrogenase activity (DHA), following different cover crop treatments: BM, bare-soil fallow followed by common bean; B1, one cycle of *U. brizantha* cover cropping before common bean; B2, two consecutive cycles of *U. brizantha* cover cropping before common bean; PB, a perennial *U. brizantha* pasture; PS, Pristine soil under native vegetation, included as a reference site. Different letters indicate significant differences among treatments (*p* < 0.05). Colors represent the different treatments: BM (red), B1 (blue), B2 (light blue), PB (light green), and PS (dark green).

Enzymatic activity differed significantly among treatments (Figure 7, Supplementary Table S3). For FDA, the PB treatment exhibited the highest activity, while B1, B2, and BM did not differ significantly from each other (Figure 7). AP activity differed significantly among treatments: PB and B1 had the highest activities and did not differ from each other, followed by B2 with intermediate values, and BM, which showed the lowest activity (Figure 7). No significant differences were observed among treatments for DHA (Figure 7).

### 3.6. The Associations Between Fungal Communities and Environmental Factors

**Figure 8:**
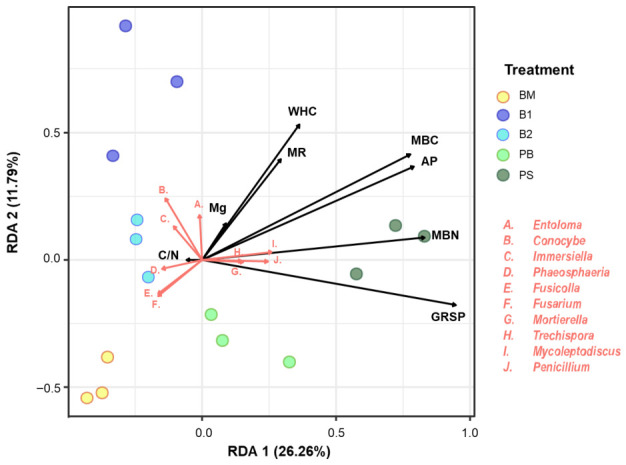
Redundancy Analysis (RDA) plot showing the relationship between fungal communities (orange arrows) and rhizosphere soil properties (black arrows) following different cover crop treatments: BM, bare-soil fallow followed by common bean; B1, one cycle of *U. brizantha* cover cropping before common bean; B2, two consecutive cycles of *U. brizantha* cover cropping before common bean; PB, a perennial *U. brizantha* pasture; PS, Pristine soil under native vegetation, included as a reference site.

Redundancy analysis (RDA) was performed to assess the influence of soil biological and physicochemical properties on microbial community composition at the genus level (Figure 8). The first axis, RDA1, explained 26.26% of the total variation, and separated treatments along a clear gradient from BM, through B1, B2, and PB towards PS. The second axis, RDA2, explained 11.79% of the variation and further distinguished BM, B1, and B2. Pristine soil (PS) samples were clearly separated from managed treatments and were positively associated with indicators of microbial biomass and activity (MBC, MBN, MR and AP), as well as with soil structural and chemical properties such as GRSP, Mg, and WHC. These variables were associated with the fungal genera *Mortierella*, *Trechispora*, *Mycoleptodiscus*, and *Penicillium*. The PB treatment occupied an intermediate position and showed associations with similar environmental variables and fungal taxa as PS. In contrast, B1 and B2 treatments were associated with the soil C/N ratio and with the genera *Entoloma*, *Conocybe*, *Immersiella* and *Phaeosphaeria*. The BM treatment clustered separately from all other treatments and was associated with *Fusicolla* and *Fusarium* genera.

Forward selection retained eight environmental variables: GRSP, MR, MBN, MBC, Mg, WHC, AP, and C:N, and all variance inflation factors remained below 10, indicating no multicollinearity issues. The constrained model was globally significant (pseudo-F = 2.34, *p* = 0.001), explaining 75.7% of the total variance in community composition (R^2^ = 0.757, adjusted R^2^ = 0.433). The first three RDA axes were significant (RDA1: F = 6.49, *p* = 0.001; RDA2: F = 2.91, *p* = 0.005; RDA3: F = 2.67, *p* = 0.028), collectively capturing the major gradients structuring the community. Marginal permutation tests revealed that GRSP (F = 2.73, *p* = 0.002), C:N (F = 1.97, *p* = 0.020), and AP (F = 1.90, *p* = 0.042) exerted the strongest independent effects on community structure. Variation partitioning further showed that soil biological properties (GRSP, MR, MBN, MBC) explained a substantially larger unique fraction of community variation (adj. R^2^ = 0.246, *p* = 0.002) than physicochemical properties (Mg, WHC, AP, C:N; adj. R^2^ = 0.125, *p* = 0.018), while the shared fraction between both sets was relatively small (adj. R^2^ = 0.062), suggesting that biological and physicochemical drivers shape microbial community assembly through largely independent mechanisms. Given the moderate sample size relative to the number of constraints, the constrained ordination is interpreted as a descriptive representation of the main gradients structuring the fungal community rather than as a predictive model; accordingly, adjusted R^2^ values [55] were used throughout to account for model complexity and avoid overfitting.

### 3.7. Correlation Analysis Between Fungal Communities and Soil Properties

**Figure 9:**
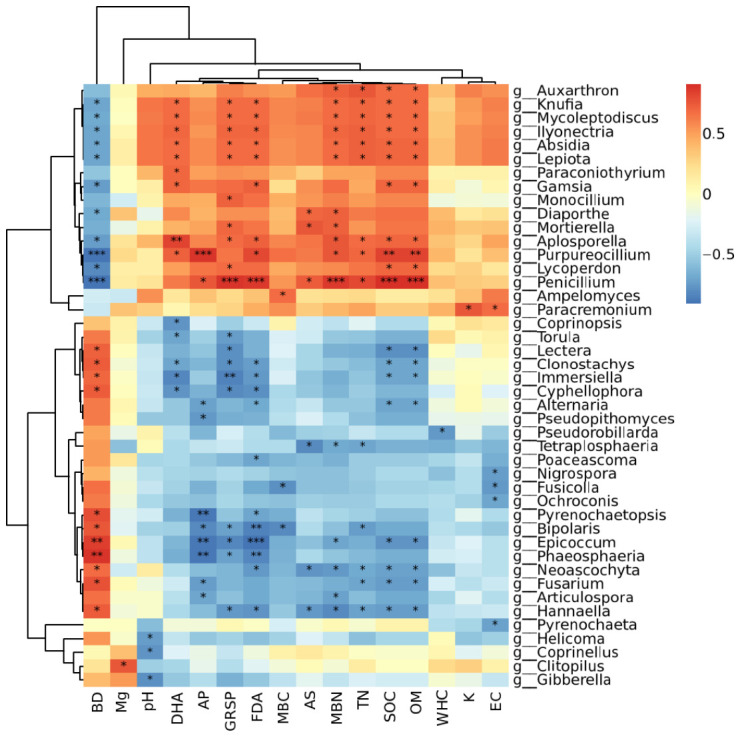
Heatmap of Spearman correlation coefficients between soil properties and fungal communities. Red indicates positive correlations and blue negative correlations. Asterisks denote significance: *: *p* ≤ 0.05, **: *p* ≤ 0.01, ***: *p* ≤ 0.001. BD: bulk density; Mg: magnesium; pH: soil pH; DHA: dehydrogenase activity; AP: acid phosphatase activity; GRSP: glomalin-related soil protein; FDA: fluorescein diacetate hydrolysis; MBC: microbial biomass carbon; AS: aggregate stability; MBN: microbial biomass nitrogen; TN: total nitrogen; SOC: soil organic carbon; OM: organic matter; WHC: water-holding capacity; K: potassium; EC: electrical conductivity.

Spearman correlation analysis revealed significant associations between fungal taxa and soil properties (Figure 9). A group of fungal taxa was positively associated with indicators of microbial activity and organic matter content, including dehydrogenase activity, fluorescein diacetate hydrolysis, microbial biomass nitrogen, soil organic carbon, organic matter, and glomalin-related soil protein, while showing negative correlations with soil bulk density. In contrast, another group of taxa exhibited the opposite pattern, being associated with higher bulk density and reduced biological activity. For example, *Epicoccum* and *Bipolaris* were positively and significant associated with FDA activity and AP correlated positively and significantly with *Epicoccum*, *Pyrenochaetopsis*, and *Phaeosphaeria*.

## 4. Discussion

### 4.1. U. brizantha Integration Shapes Fungal Communities in the Common Bean Rhizosphere

Alpha diversity metrics (Shannon, Pielou’s evenness, observed features) did not differ significantly among treatments, indicating that management practices did not alter the overall taxonomic diversity of fungal communities. The decoupling between alpha and beta diversity metrics provides critical insights into the ecological assembly mechanisms driving these rhizosphere legacy effects. The lack of significant differences in fungal richness and evenness suggests that the inclusion of *U. brizantha* during fallow periods did not lead to a simplistic reduction or expansion of the available ecological niches. Instead, beta diversity analyses revealed a clear differentiation in community composition across treatments (Figure 2), demonstrating a profound process of species turnover. This turnover reflects a targeted ecological restructuring driven by changes in the quantity and quality of belowground carbon inputs from the cover crop roots, as well as shifts in soil microenvironmental conditions. In this context, fungal communities shifted from degraded monoculture toward a structure more similar to that observed in systems with continuous living roots. Specifically, this mechanism allowed the selective recruitment of specialized fungal guilds while displacing others, leading to the suppression of persistent, host-specific agricultural pathogens, such as *Fusarium* and *Fusicolla*, which typically accumulate during extended monocultures. Simultaneously, it fostered the establishment of robust saprotrophic and biological control agents like *Mortierella* and *Penicillium*. Previous studies in common bean systems have similarly shown that sustainability-oriented practices, such as the inclusion of cover crops, promote functionally complex fungal communities [56]. Therefore, the ecological legacy of *U. brizantha* in these degraded agroecosystems is characterized not by a quantitative expansion of taxonomic richness, but by a qualitative and functional reorganization of the rhizosphere mycobiome toward a more resilient state that influences community structure even after the reintroduction of the cash crop.

The dominance of *Ascomycota*, *Mortierellomycota*, and *Basidiomycota* across all treatments is consistent with previous reports in agricultural soils [57,58,59]. However, clear shifts at the genus level reflected differences among management regimes. The common bean monoculture was characterized by higher relative abundances of *Fusarium* and *Fusicolla*—genera that include plant pathogens—suggesting that continuous monocropping may favor fungal groups potentially associated with increased disease risk [60,61]. Similar patterns have been reported in simplified agroecosystems, where reduced plant diversity can facilitate pathogen accumulation, whereas diversified systems promote beneficial microbial communities and greater overall microbial stability [56,62,63,64].

In contrast, the inclusion of *U. brizantha* promoted greater representation of saprotrophic genera, including *Mortierella*, *Penicillium*, and *Coprinellus*, taxa frequently associated with organic matter turnover and nutrient mobilization [22,65]. When compared with alternative grass cover crops such as ryegrass or cereal rye, which typically induce more transient fungal shifts due to their softer and rapidly decomposing tissues, the persistent legacy of *U. brizantha* appears to be driven by its high C ratio and lignified root architecture. This recalcitrant biomass acts as a long-term specialized substrate that selectively favors saprotrophic fungal communities, consistent with observations from tropical maize–Urochloa intercropping systems where specific varieties exert prolonged selective pressure on soil fungal networks [22]. The enrichment of these fungi is particularly relevant because saprotrophic taxa play a key role in residue decomposition and nutrient-release processes, thereby contributing to soil quality restoration and chemical recovery in degraded agroecosystems [66]. Although these groups are functionally diverse and their ecological roles may vary considerably among species and strains, several members are known to produce metabolites that enhance plant resilience under environmental stress [67]. Therefore, the sustained enrichment of these fungal taxa may indicate a shift toward microbial communities capable of simultaneously supporting nutrient cycling and improving plant tolerance to stressful conditions, although strain-level analyses are required to confirm the underlying functional mechanisms. *Penicillium* species, besides acting as saprotrophs, contribute to plant nutrition and health through the production of solubilized phosphorus, siderophores, and phytohormones [68,69]. Likewise, *Mortierella* species are capable of degrading complex carbon polymers and improving access to bioavailable P and Fe, even under unfavorable conditions, thereby supporting plant resilience [70]. The increase in acid phosphatase activity in the *U. brizantha* treatments indicates enhanced biological P mobilization [71]. However, the absence of differences in extractable P, even after long-term management, suggests that mobilized P is rapidly taken up by plants or immobilized within the microbial biomass [72].

This pattern is likely reinforced by the high P demand of common bean, particularly considering that soil sampling was conducted during the reproductive stage of the crop, when nutrient uptake is maximal.

### 4.2. Biomarker Taxa Revealed by LEfSe Analysis

The LEfSe analysis revealed distinct fungal phyla associated with each management practice. The common bean monoculture (BM) was characterized by *Ascomycota* and genera such as *Fusarium*, *Fusicolla*, and *Bipolaris*, indicating a community enriched in taxa that includes well-known plant pathogens. Among these, the enrichment of *Fusicolla* in BM was confirmed by the conservative ZIGMM analysis, whereas *Fusarium*, despite its high relative abundance, was not statistically supported under this more stringent model, likely reflecting its high inter-replicate variability. This pattern is consistent with previous reports showing that continuous monoculture can favor pathogenic fungi and reduce microbial functional balance [73,74]. In contrast, one and two cycles of *U. brizantha* were associated with saprotrophic and potentially beneficial genera, including *Immersiella*, *Clonostachys*, *Torula*, *Lectera*, *Alternaria*, and *Psathyrella*, suggesting an enhancement of decomposition processes and nutrient cycling in response to pasture cover cropping. Cover crops have been shown to enhance saprotrophic fungal communities involved in residue breakdown and nutrient cycling, thereby increasing soil functional capacity [57]. Perennial *U. brizantha* (PB) was enriched in *Gamsia*, *Chaetomium*, and *Pyrenochaeta*, taxa associated with soil stability and biocontrol, indicating that longer-term cover crop management may foster a more resilient fungal community [75,76]. Finally, pristine soil (PS) harbored *Penicillium*, *Mycoleptodiscus*, and *Purpureocillium*, reflecting a fungal community structure characteristic of undisturbed systems. Overall, these findings reinforce that cover crop rotations can substantially modulate rhizosphere fungal communities, shifting them away from pathogen-enriched assemblages typical of monoculture toward saprotroph-dominated and potentially more functionally resilient configurations.

### 4.3. Functional Guilds of the Rhizosphere Fungal Community

The functional guild analysis suggests that soil management history influences the balance of fungal ecological roles by modifying plant residue inputs, root activity, and soil biological conditions. These factors likely shape the relative abundance of saprotrophic, pathogenic, and endophytic fungi in the rhizosphere. In particular, the monoculture system (BM) showed a marked increase in plant pathogenic fungi, consistent with previous studies indicating that continuous monocropping promotes pathogen accumulation, decline of beneficial microbes, and microbial imbalance, which together drive disease and yield loss [77]. In contrast, the B1 treatment showed a shift toward a more regulated functional profile, with lower representation of pathogenic guilds while maintaining saprotrophic activity, indicating that using cover crops can help disrupt pathogen cycles and promote more balanced plant–soil interactions [78]. This shift suggests that diversification practices may contribute to enhancing rhizosphere resilience and reducing the risk of disease development in cropping systems. However, these functional shifts must be interpreted with caution, as FUNGuild outputs are predictive models dependent on current database annotations rather than direct in situ metabolic measurements.

### 4.4. Effects of U. brizantha in Soil Properties and Enzymatic Activities

The inclusion of *U. brizantha* markedly influenced soil structural and biological indicators compared with the common bean monoculture, which consistently showed the lowest values across most variables. Higher SOC and organic matter levels under *U. brizantha*, particularly in the B2 and perennial pasture treatments suggest greater organic inputs and enhanced C stabilization, likely associated with sustained root biomass, rhizodeposition, and reduced soil disturbance [23,24]. These changes were accompanied by lower bulk density and higher aggregate stability, indicating improved soil structural resilience [79]. Tropical grasses with dense root systems are widely recognized for promoting aggregation through both physical root entanglement and microbial-mediated binding processes, reinforcing the role of *U. brizantha* in enhancing soil physical quality.

Importantly, the response followed a management gradient. While one or two cover crop cycles (B1 and B2) already promoted improvements relative to monoculture, the perennial pasture showed the strongest effects, approaching values observed in the reference site. This pattern suggests that both the presence and the duration of plant cover are key drivers of soil structural recovery [80]. However, although perennial *U. brizantha* maximized soil improvements, maintaining consecutive years without a cash crop may represent a practical limitation for grain-oriented systems. In this context, the intermediate responses observed under B1 and B2 indicate that shorter-term integrations may provide a realistic compromise, achieving substantial gains in soil quality while preserving production feasibility.

Microbial respiration, microbial biomass C and N, and GRSP were generally higher under *U. brizantha* treatments, particularly in PB, indicating enhanced microbial activity. Together with the observed shifts in fungal community composition, these results suggest that the inclusion of *U. brizantha* not only restructures fungal assemblages but also strengthens their functional contribution to soil processes related to nutrient cycling and aggregation [22,81].

### 4.5. Relationships Between Soil Properties and Fungal Communities

The RDA revealed a clear gradient in fungal community structure associated with management intensity, ranging from the bare-soil fallow system toward the pristine soil condition, indicating a consistent response of fungal community assembly to management practices. Along this gradient, treatments progressively shifted toward conditions characterized by higher microbial biomass, respiration, and improved soil structural properties. This pattern suggests that soil management practices strongly influence rhizosphere fungal communities by modifying the biological functioning of the soil environment [75]. In particular, the results suggest that soil biological processes are primary determinants of fungal community structure, as variation partitioning showed that biological variables (GRSP, MBC, MBN, and MR) explained a larger unique fraction of community variation than physicochemical properties [81]. GRSP emerged as a key driver of fungal community organization, likely due to its role in promoting aggregate stability and facilitating hyphal network development [82,83,84]. Since GRSP is closely associated with fungal biomass production and soil structural maintenance, this result suggests that management practices capable of stimulating fungal-mediated carbon stabilization may contribute to the reorganization of rhizosphere communities. Importantly, the incorporation of *Urochloa brizantha* cover cropping shifted fungal community composition along the ecological recovery gradient. Even a single cover crop cycle (B1) produced detectable changes in community structure, indicating that implementation of one cycle of cover crop can initiate soil biological restoration processes. The association of B1 and B2 with higher C:N ratios suggests that residue quality and nutrient stoichiometry may regulate transitional stages of microbial community restructuring. These patterns collectively indicate that cover cropping does not merely increase fungal diversity but promotes a functional reorganization of the rhizosphere microbiome toward communities associated with organic matter turnover, structural stabilization, and nutrient cycling [85,86,87]. Furthermore, the legacy effects driven by *U. brizantha* mirror the environmental benefits achieved through other advanced sustainability interventions. For instance, recent frameworks emphasize that the deployment of ecological restoration tools and structured amendments not only enhances fertility but also successfully redirects microbial metabolic networks toward functional optimization [88,89]. Our findings reinforce that such legacy effects operate through both biological and physicochemical pathways—integrating changes in microbial activity, residue inputs, and soil structure to drive long-term shifts in fungal community assembly. Overall, this demonstrates that even short-term cover crop implementation can rapidly influence rhizosphere functioning, initiating ecological recovery trajectories in degraded agroecosystems and supporting their integration as a sustainable soil management strategy.

### 4.6. Correlation Analysis

Correlation analysis indicated that specific fungal taxa were associated with fertile and biologically active soils. The positive relationships with enzymatic activities (DHA, FDA, AP), microbial biomass (MBN), and nutrient pools (SOC, TN, OM) suggest that these taxa are favored in soils with enhanced organic matter turnover and nutrient cycling [78,82]. The negative correlations with bulk density indicate an association with less compacted soils, characterized by greater porosity and more favorable conditions for microbial activity [80]. Genera such as *Penicillium* and *Purpureocillium* have been reported to exhibit saprophytic and nutrient-solubilizing capacities [69,90], while *Gamsia* and *Knufia* has been associated with stress tolerance and organic matter decomposition [91]. In this context, the observed associations are consistent with a role of these taxa in organic matter turnover and nutrient cycling.

Positive associations of *Knufia*, *Aplosporella*, *Purpureocillium*, and *Penicillium* with enzymatic activity, nutrient availability, and soil organic matter indicate that these taxa may contribute to nutrient cycling, organic matter stabilization, and overall soil fertility, in line with their reported saprotrophic or plant growth-promoting functions [69,91]. Conversely, negative correlations of *Fusarium*, *Clonostachys*, *Alternaria*, and *Bipolaris* with soil enzymatic activity and nutrient pools, together with their positive association with bulk density, suggest that these taxa may proliferate under soil compaction, reduced aeration, and lower organic matter content, conditions often linked to soil degradation and plant stress [92,93]. These patterns are consistent with the notion that management practices enhancing organic matter inputs and enzymatic activity can shift fungal communities toward beneficial taxa while suppressing opportunistic or pathogenic groups, thereby improving soil resilience and agroecosystem sustainability.

## 5. Conclusions

The integration of *Urochloa brizantha* into common bean systems reshaped rhizosphere fungal community composition without affecting overall diversity, indicating that management primarily drives species turnover rather than richness. Cover crop inclusion shifted communities away from pathogen-enriched assemblages typical of monoculture toward more functionally balanced configurations associated with enhanced soil biological activity. Importantly, even a single cover crop sequence was sufficient to induce these shifts. While long-term functional resilience, disease suppression, and crop productivity remain to be directly measured, these results demonstrate that interrupting monoculture through strategic cover crop inclusion can significantly stimulate soil biological functioning without compromising cropping frequency, supporting potentially more sustainable and agronomically viable production systems.

## Data Availability

The original contributions presented in this study are included in the article/Supplementary Materials. Further inquiries can be directed to the corresponding authors. Please be informed that the high-throughput sequencing data have been uploaded to the NCBI database, under BioProject ID: PRJNA1459626. You can access the data through the following link: https://www.ncbi.nlm.nih.gov/bioproject/PRJNA1459626 (accessed on 16 Febrery 2026).

## Supplementary Materials

The following supporting information can be downloaded at: https://www.mdpi.com/article/10.3390/jof12070456/s1, Figure S1: Diversity indices of fungal communities in rhizosphere soils: (A) Shannon, (B) Pielou’s eveness, (C) Observed features, measured in the rhizosphere of the following treatments (n = 3): CB = common bean monoculture; B1 = *U. brizantha*/common bean; B2 = *U. brizantha*/*U. brizantha*/common bean; PB = perennial *U. brizantha*; PS = Pristine soils under native vegetation, included as a reference site. Different lowercase letters indicate statistically significant differences among treatments based on ANOVA followed by Tukey’s HSD test (*p* ≤ 0.05); Supplementary Table S1: Genus-level differential abundance of rhizosphere fungal communities; Supplementary Table S2: Trimming summary showing the number of initial reads, reads that pass quality control, reads after denoising step, number of sequences after merging them by their 3′, and the resulting non-chimeric sequences. CB = common bean monoculture; B1 = *U. brizantha*/common bean; B2 = *U. brizantha*/*U. brizantha*/common bean; PB = perennial *U. brizantha*; PS = Pristine soils under native vegetation, included as a reference site. Supplementary Table S3: Mean values of chemical, physical and microbiological soil properties in the rhizosphere soil (0–10 cm depth) of common bean across treatments: BM, bare-soil fallow followed by common bean; B1, one cycle of *U. brizantha* cover cropping before common bean; B2, two consecutive cycles of *U. brizantha* cover cropping before common bean; PB, a perennial *U. brizantha* pasture; PS, Pristine soils under native vegetation, included as a reference site. In each row, different upper-case letters indicate significant differences (*p* ≤ 0.05) between treatments according to LSD’s test.
