# Supplementary material for: Legacy Effects of Urochloa brizantha Cover Cropping on Rhizosphere Fungal Communities and Soil Properties in a Degraded Common Bean System
Source: J Fungi (Basel). 2026 Jun 23;12(7):456. doi: 10.3390/jof12070456 (PMC13412720; doi:10.3390/jof12070456)

Supplementary Material

Table S2. Trimming summary showing the number of initial reads, reads that pass quality control, reads after denoising step, number of sequences after merging them by their 3', and the resulting non-chimeric sequences. CB = common bean monoculture; B1 = *U. brizantha*/common bean; B2 = *U. brizantha*/*U. brizantha*/common bean; PB = perennial *U. brizantha*; PS = Pristine soils under native vegetation, included as a reference site.

Sample ID	Input	Primer-removed	Filtered	Denoised	Non-chimeric
B1R1	10067	9591	8547	7841	7829
B1R2	14204	13515	12169	11389	11104
B1R3	5628	5368	4832	4449	4446
B2R1	12803	12252	10461	9691	9686
B2R2	13209	12658	11441	10463	10455
B2R3	7897	7536	6772	6281	6281
CBR1	5313	5033	4256	3508	3504
CBR2	17784	16978	15261	13540	13381
CBR3	11208	10731	9492	8406	8357
PBR1	6178	5845	5145	4469	4462
PBR2	13773	13087	10688	9828	9782
PBR3	11054	10594	8342	7602	7600
PS1	16012	15351	12395	11165	11158
PS2	9683	9252	8138	6875	6866
PS3	10876	10345	9008	7896	7894
Total	165689	158136	136947	123403	122805

Table S3. Mean values of chemical, physical and microbiological soil properties in the rhizosphere soil (0–10 cm depth) of common bean across treatments: BM, bare-soil fallow followed by common bean; B1, one cycle of *U. brizantha* cover cropping before common bean; B2, two consecutive cycles of *U. brizantha* cover cropping before common bean; PB, a perennial *U. brizantha* pasture; PS, Pristine soils under native vegetation, included as a reference site. In each row, different upper-case letters indicate significant differences (p ≤ 0.05) between treatments according to LSD's test.

	BM		B1		B2		PB		PS
Chemical									
**SOC**	**0.99 ± 0.18**	**b**	**1.24 ± 0.12**	**ab**	**1.42 ± 0.04**	a	1.5 ± 0.06	a	2.46 ± 0.37
**OM**	**1.7 ± 0.32**	**b**	**2.13 ± 0.2**	**ab**	**2.44 ± 0.07**	a	2.59 ± 0.11	a	4.25 ± 0.64
**TN**	**0.09 ± 0.02**	**a**	**0.11 ± 0**	**a**	**0.12 ± 0.01**	a	0.12 ± 0.01	a	0.22 ± 0.04
**C.N**	**10.67 ± 0.58**	**a**	**11 ± 1**	**a**	**12 ± 0**	a	11.33 ± 0.58	a	11 ± 0
**eP**	**23.33 ± 3.79**	**a**	**22 ± 1.73**	**ab**	**20.33 ± 5.03**	ab	12 ± 1.73	b	21 ± 6

Na^{+}	0.3 ± 0	a	0.37 ± 0.06	a	0.33 ± 0.06	a	0.3 ± 0	a	0.3 ± 0
K^{+}	0.74 ± 0.12	a	0.84 ± 0.03	a	0.84 ± 0.11	a	0.7 ± 0.08	a	1.01 ± 0.18
Ca^{2+}	3.07 ± 1.07	a	4.27 ± 0.57	a	3.83 ± 0.32	a	3.43 ± 0.35	a	8.67 ± 2.08
Mg^{2+}	1.7 ± 0.35	a	1.9 ± 0.2	a	1.93 ± 0.06	a	1.83 ± 0.15	a	1.97 ± 0.38
Physical									
**WCH**	**28 ± 2.65**	**a**	**32.67 ± 1.53**	a	**31.33 ± 1.15**	a	30.67 ± 0.58	a	32.67 ± 2.08
**pH**	**7.07 ± 0.06**	**a**	**7.07 ± 0.38**	a	**6.83 ± 0.06**	a	6.93 ± 0.06	a	7.57 ± 0.21
**EC**	**0.27 ± 0.08**	**b**	**0.65 ± 0.23**	a	**0.37 ± 0.02**	b	0.25 ± 0.06	b	0.83 ± 0.22
**BD**	**1.66 ± 0.11**	**a**	**1.42 ± 0.04**	b	**1.36 ± 0.07**	b	1.29 ± 0.05	b	0.87 ± 0.18
**AS**	**14.75 ± 1.47**	**c**	**36.99 ± 8.17**	b	**50 ± 1.93**	a	44.67 ± 5.13	ab	52.33 ± 1.53
Biological									
**MR**	**0.67 ± 0.02**	**b**	**0.86 ± 0.01**	**a**	**0.9 ± 0.03**	a	0.91 ± 0.03	a	0.85 ± 0.08
**MBC**	**49.51 ± 2.9**	**b**	**72.26 ± 3.68**	**a**	**56.4 ± 0.54**	ab	62.57 ± 9.86	ab	96.33 ± 23.61
**MBN**	**13.93 ± 0.71**	**b**	**17.05 ± 1.7**	**ab**	**20.57 ± 0.9**	a	21.33 ± 3.51	a	63.56 ± 20.25
GRSP	2.3 ± 0.02	b	2.23 ± 0.24	b	2.42 ± 0.05	ab	2.68 ± 0.06	a	3.17 ± 0.16
**FDA**	**50.64 ± 0.25**	**b**	**54.91 ± 0.82**	**b**	**55.52 ± 1.87**	b	74.51 ± 5.95	a	114.52 ± 11.38
**DHA**	**8.49 ± 0.93**	**a**	**8.29 ± 0.96**	**a**	**8.91 ± 2.4**	a	9.88 ± 0.48	a	12.7 ± 1.05
**AP**	**8.01 ± 0.34**	**c**	**14.9 ± 1.76**	**a**	**11.86 ± 0.51**	b	16.29 ± 0.55	a	18.72 ± 2.97

Figure S1. Diversity indices of fungal communities in rhizosphere soils: A) Shannon, B) Pielou´s eveness, C) Observed features, measured in the rhizosphere of the following treatments (n = 3): CB = common bean monoculture; B1 = *U. brizantha*/common bean; B2 = *U. brizantha*/*U. brizantha*/common bean; PB = perennial *U. brizantha*; PS = Pristine soils under native vegetation, included as a reference site. Different lowercase letters indicate statistically significant differences among treatments based on ANOVA followed by Tukey's HSD test ($p \leq 0.05$).

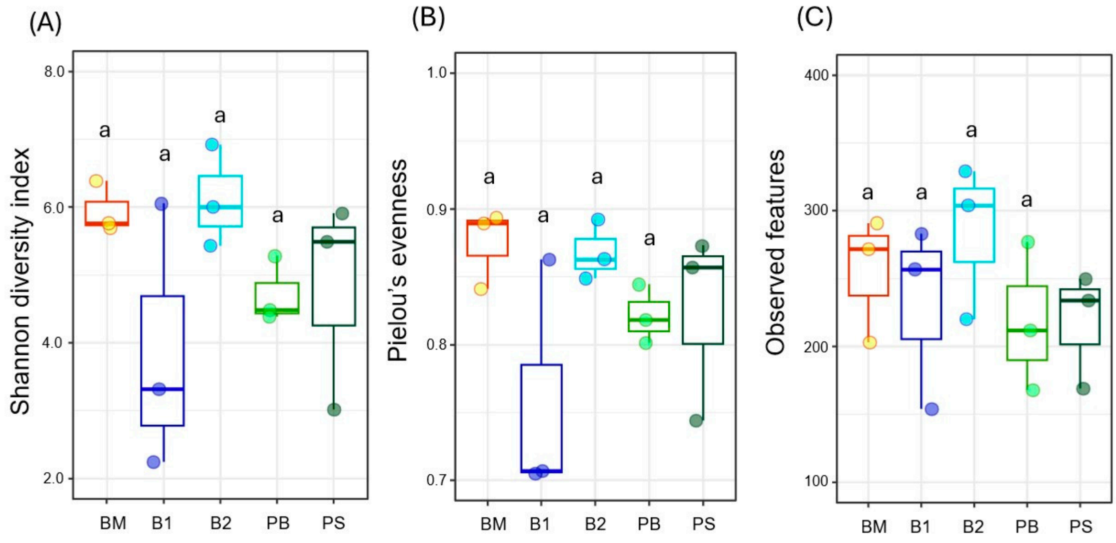

Supplement: Supplementary file 1 [file jof-12-00456-s001.zip › TableS2,S3 and FigureS1.pdf]
